# Pulmonary changes in Norwegian fatal cases of pandemic influenza H1N1 (2009) infection: a morphologic and molecular genetic study

**DOI:** 10.1111/irv.12410

**Published:** 2016-09-01

**Authors:** Pål Voltersvik, Lara A. Aqrawi, Susanne Dudman, Olav Hungnes, Norwegian Lung Pathology Group, Leif Bostad, Karl A. Brokstad, Rebecca J. Cox, Erik Heyerdahl Strøm, Torleiv O. Rognum, Jan Mæhlen, Glenny Cecilie Alfsen, Trond Viset, Ingjerd Lien Kvelstad, Inge Morild

**Affiliations:** ^1^ The Influenza CentreDepartment of Clinical ScienceUniversity of BergenBergenNorway; ^2^ K. G. Jebsen Centre for Influenza Vaccine ResearchUniversity of BergenBergenNorway; ^3^ WHO National Influenza CentreNorwegian Institute of Public HealthOsloNorway; ^4^ Gade Laboratory for PathologyDepartment of Clinical MedicineHaukeland University HospitalUniversity of BergenBergenNorway; ^5^ The Broegelmann Research LaboratoryDepartment of Clinical ScienceUniversity of BergenBergenNorway; ^6^ Department of Research and DevelopmentHaukeland University HospitalBergenNorway

**Keywords:** 2009 pandemic, HA pyrosequencing, immunohistochemistry, influenza, lung

## Abstract

**Background:**

During the pandemic outbreak of the 2009 swine influenza (A(H1N1)pdm09), 32 fatal cases occurred in Norway and 19 of these were included in this study.

**Objectives:**

We characterised pulmonary changes in these fatal Norwegian cases.

**Patients and Methods:**

Upon hospitalisation, detailed clinical information and specimens from the upper and lower respiratory pathways were collected. At post‐mortem, lung tissue was collected, formalin‐fixed and paraffin‐embedded. Immunohistochemical and light microscopic examination was performed to visualise the local expression of the A(H1N1)pdm09 virus. Reverse transcription‐polymerase chain reaction (RT‐PCR) and pyrosequencing of the non‐fixed specimens allowed the identification of mutations in the influenza virus surface glycoprotein (haemagglutinin gene) particularly at position 222.

**Results and Conclusions:**

The overall course of illness lasted from 2 to 40 days (median 9 days). Diffused alveolar damage (DAD) was evident in 11 cases, 4 of which had no apparent underlying illness. Obesity was prominent in 12 cases, where three individuals were classified as otherwise healthy. The HA D222G mutation was detected in six cases, 3 of which had no underlying illness. Immunohistochemistry showed the A(H1N1)pdm09 virus to be prominent at the site of inflammation both in close proximity to and inside alveolar structures in the lung tissue. In addition to a possible role for the HA D222G mutation, our findings indicate that host factors and underlying conditions in the infected individuals are fundamental for disease outcome in many cases. This study increases our understanding of determinants for the clinical outcome of pandemic influenza, which could guide future treatment.

## 1 Introduction

In April 2009, an outbreak of a novel influenza A virus of swine origin was discovered in Veracruz, Mexico, subsequently referred to as influenza A(H1N1)pdm09.^1^, ^2^ In the following months, the virus rapidly spread worldwide causing the World Health Organization (WHO), on 11 June 2009, to declare the first influenza pandemic of the 21st century. During the first wave of the 2009 pandemic, Norway, with a population of 4.9 million people,^3^ was comparably well prepared for the ongoing pandemic.^4^, ^5^, ^6^, ^7^, ^8^, ^9^, ^10^ Norway had access to a pandemic vaccine and mass vaccination started 1–3 weeks prior to the peak of the pandemic, resulting in high vaccination coverage (45%) and reduced morbidity and mortality.^11^ Emergency plans were in place, but fortunately the high number of hospitalisations which were planned for in the reasonable worst case scenario did not occur. In total, 1300 people diagnosed with influenza were hospitalised, 200 patients received intensive care, and 32 patients died with laboratory‐verified infection.^12^, ^13^ Despite being described as a relatively mild pandemic, some young healthy individuals experienced severe illness and occasional mortality.^14^, ^15^, ^16^, ^17^, ^18^ Interestingly, elderly patients (>65 years old) were much less severely affected, probably due to pre‐existing cross‐reactive immunity generated by infection with similar H1N1 viruses in early life.^19^

Post‐pandemic studies have described the clinical aspects of the A(H1N1)pdm2009, where several host factors and underlying conditions, such as pneumonia, obesity and pregnancy, have been associated with disease severity.^9^, ^14^, ^15^, ^16^, ^20^, ^21^, ^22^ Also, the impact of vaccines and neuraminidase inhibitors used during the pandemic has been investigated.^23^, ^24^, ^25^ Interestingly, viral factors, such as mutations altering the receptor‐binding specificity of the virus, have been identified and associated with severe disease outcome.^26^, ^27^, ^28^, ^29^, ^30^ Moreover, the most severe clinical cases from the A(H1N1)pdm09 have been shown to relate to the severity of lung pathological damage, as observed previously in the highly virulent 1918 pandemic and in zoonotic avian H5N1 cases. In addition to inflammation of the larger airways, these viral infections involve the alveolar walls and may cause diffused alveolar damage (DAD) in susceptible individuals.

In the current study, we performed a clinicopathological analysis of 19 fatal, autopsied Norwegian cases from the 2009 pandemic. We amplified the influenza virus haemagglutinin (HA) gene to identify possible genetic viral mutations and visualised the expression of the A(H1N1)pdm09 virus inflammation in the lung tissue of autopsied subjects. Our results show a possible association between obesity, pre‐existing illness, the viral mutation HA D222G and mortality in fatal Norwegian cases.

## 2 Materials and Methods

### 2.1 Study population

Nineteen hospitalised fatal autopsied cases with laboratory‐verified A(H1N1)pdm09 infection (13 males and 6 females, aged 9–69 years old) were included in our study. Patients were admitted to clinical wards at Oslo University Hospital, Akershus county, Lillehammer, Kristiansand, Haukeland University Hospital in Bergen and St. Olav Hospital in Trondheim. Two cases were sudden, unexpected deaths that occurred outside hospital, and according to regulation reported to the police who ordered forensic autopsy. Clinical and demographic information (Table 1) and also respiratory tract and lung swabs were collected upon hospitalisation. In addition, lung/bronchial tissue was collected post‐mortem and examined by an experienced pulmonary pathologist. The Regional Ethics Committee of Western Norway approved this study and the use of samples without patient consent (REC2009/1224).

**Table 1:** Patients' demographics

Patient	Gender	Age (y)	Weight (kg)	BMI	Obesity	Pre‐existing disease	Duration of illness (d)	Lung tissue patterns	Neuraminidase inhibition therapy
1	M	50–59	110	32	+	Healthy	7	Virus pneumonia. HM	−
2	F	40–49	128	48	+	Asthma	7	Bacterial pneumonia	+
3	F	11–20	–	–	−	Congenital cardiomyopathy	9	Virus pneumonia	+
4	M	20–29	121	35	+	Healthy	40	Virus pneumonia. HM. DAD	+
5	F	1–10	–	–	−	Eisenmenger syndrome/Trisomia	8	Virus pneumonia. HM. DAD	−
6	M	11–19	37	17	−	Autoimmune disease/cancer	2	Bacterial pneumonia	−
7	F	30–39	148	53	+	Healthy	11	Virus pneumonia. HM. DAD	−
8	M	20–29	115	37	+	Healthy	10	Virus pneumonia. HM. DAD	−
9	F	60–69	73	31	+	Asthma/Rheumatoid Arthritis	12	Mixed viral and bacterial infection. DAD. ARDS	+
10	M	50–59	108	35	+	Arteriosclerosis	35	Virus pneumonia. HM. DAD	+
11	F	50–59	114	39	+	Myelomatosis	14	Virus pneumonia. HM	−
12	M	40–49	–	–	−	Healthy	3	Non‐specific (not characteristic for infection)	+
13	M	30–39	58	23	−	Healthy	7	Virus pneumonia. HM. DAD	+
14	M	40–49	85	25	−	Chronic lymphatic leukaemia	20	Virus pneumonia. DAD. ARDS	−
15	M	50–59	90	31	+	Hypertension	10	Virus pneumonia. HM. DAD	−
16	M	30–39	98	36	+	Pancreatitis/alcoholic liver disease	31	Fungus pneumonia (Aspergillus)	+
17	M	30–39	79	29	−	Healthy	2	Virus pneumonia. HM	−
18	M	40–49	97	29	−	Hypertension/Diabetes II	3	Virus pneumonia. HM. ARDS	−
19	M	50–59	73	24	−	Chronic lymphatic leukaemia	7	Virus pneumonia. HM. DAD	+

M, male; F, female; BMI, body mass index; HM, hyaline membranes; DAD, diffused alveolar damage; ARDS, acute respiratory distress syndrome.

### 2.2 Sequence analysis

Viral RNA was extracted using a total nucleic acid extraction kit in the MagNA Pure LC System (Roche Diagnostics, Mannheim, Germany). In general, a modification of the full genome sequencing protocol provided by CDC, USA,^31^ was used for virus isolates, whereas sequencing of the amplicon from a more sensitive RT‐PCR^32^ was used for many of the primary specimens.

A pyrosequencing assay was used to detect D222G mutations in the HA1 gene in the Norwegian A(H1N1)pdm09 cases as described previously.^30^ Briefly, a 110‐nucleotide amplicon encompassing the HA 222 amino acid region was generated from 5 μL specimen RNA combined with each of primers pyro‐H1 forward: 5′‐AGTTCAAGCCGGAAATAGCA‐3′ and pyro‐H1 reverse: 5′‐biotin‐TTTCCAGTTGCTTCGAATGTT‐3′ and reagents from the SuperScript III One‐Step RT‐PCR System with Platinum Taq High Fidelity kit (ThermoFisher Scientific, Waltham, MA, USA) in a 25 μL reaction and subjected to the following cycling conditions: 30 minutes at 50°C, 2 minutes at 94°C; 45 cycles of 15 seconds denaturation at 94°C, 30 seconds annealing at 55°C and 1 minute extension at 68°C; finally, 5 minutes at 68°C for final extension.

The pyrosequencing reactions were performed as previously described^33^ using a residue‐specific sequencing primer (5′‐AGCAATAAGACCCAAAGTGAGG‐3′). The sequenced region begins at nucleotide 747 in the full‐length sequence of the viral RNA segment 4, where nucleotides 747–749 encode HA1 amino acid 222. The most common wild‐type codon in this position is GAT, which encodes aspartic acid (D). The GAA codon encodes glutamic acid (E), while GGT (or GGA) gives glycine (G). Mutation of G to A in the first position of the GAT codon gives asparagine (N).

### 2.3 Immunohistochemistry

Routine haematoxylin–eosin‐stained sections of formalin‐fixed, paraffin‐embedded lung tissue were prepared according to the standard protocols.

Immunohistochemical staining was performed by the EnVision technique (K5007, Dako, Glostrup, Denmark) following the manufacturer's instructions, and 1:1000 dilution of anti‐influenza A matrix protein (E10) antibody (#EMS009, KeraFAST, Boston, MA, USA).^34^, ^35^

### 2.4 Light microscopy and evaluation of immunohistochemical staining

All sections were studied using a light microscope (Leica DMLB, Leica Microsystems Wetzlar, Germany) by three investigators. Sections were scored blindly by two investigators to assess the degree of A(H1N1)pdm09 staining in the lung tissue. Depending on the degree of positivity, the number 0, 1 or 2 was assigned for each category during assessment, where 0 was considered negative, 1 was regarded positive and 2 represented strongly positive. As two sections were evaluated from each subject, the mean score value from both sections was calculated for each individual. Cells were considered positive when ≥50% of the cell membrane was positively stained by antibody.

### 2.5 Statistical significance

Statistical significance was evaluated by the Student *t* test and presented as the mean. Differences were considered significant when *P*≤.05. In addition, the Pearson correlation test was used to examine the association between the different parameters.

## 3 Results

### 3.1 Obesity and pre‐existing illness as contributors to disease severity in the 2009 pandemic influenza A H1N1 fatal Norwegian cases

The nineteen fatal cases that were hospitalised during the 2009 pandemic in Norway consisted of 13 males and 6 females, aged 9–69 years old. Generally, the course of disease lasted 2–40 days. In 15 individuals (79%), the course of infection was ≤14 days. Moreover, neuraminidase inhibitors (laninamivir/oseltamivir (Tamiflu) 75 mg/peramivir/zanamivir (Relenza)) were administered to nine patients (47%). Underlying disease was observed in 12 of the cases (63%). Three of the 7 previously healthy patients (six males and one female) received neuraminidase inhibitors.

Light microscopic examination of the lung tissue revealed that 14 patients had viral pneumonia, of which 11 showed hyaline membranes. In the remaining five patients, two had bacterial infection, one had a mixed viral and bacterial infection, one had fungal infection (aspergillus), and one had no apparent infection in the tissue (Table 1). Interestingly, post‐mortem examination of the lung tissue revealed that DAD was evident in 11 individuals (78%) (eight males) with 4 of these had DAD, but no apparent pre‐existing illness. Three patients had acute respiratory distress syndrome pattern (ARDS), 2 of these patients showed changes consistent with viral pneumonia and 1 patient most likely had a mixed viral and bacterial infection.^36^ None of the three patients with clinical ARDS showed DAD morphologically. Meanwhile, 2 had only viral infection and 1 demonstrated a mixed viral and bacterial infection.

Obesity has been shown to be a prominent contributor to disease severity during the pandemic. Twelve patients (eight males and four females, ages 27–69 years) had body mass index (BMI) ranging from 29 to 53. The overall course of illness ranged from 2 to 40 days (median 9 days), while in the obese cases the range was 7–40 days, with a slightly longer median of 12 days. Interestingly, 5 of 7 individuals with no known pre‐existing illness were obese. In addition to this, 6 of the obese cases had DAD in their lung tissue (Table 1).

### 3.2 A Haemagglutinin viral mutation (HA D222G) in A(H1N1)pdm09 is associated with severe disease outcome

Swabs from the upper and lower respiratory tract were collected from 15 of the fatal cases allowing genetic analysis of the viral haemagglutinin gene, using conventional (Sanger) sequencing or pyrosequencing. The viral genotype at amino acid position 222 of the haemagglutinin (HA) gene was determined in 15 individuals. The HA substitution D222G was detected in six cases (four males and two females, aged 25–59 years), while the other nine patients possessed the wild‐type D222D. Interestingly, disease duration was ≤14 days in 5 of the subjects possessing the HA mutation, while obesity was prominent in 4 of the cases. No apparent association between swab location and detection of the HA D222G mutation was observed in these subjects (Table 2).

**Table 2:** Patients' qPCR values for H1N1

Patient	Gender	Age (y)	Influenza A	H1N1	Ct value	Mutation	Swab location
1	M	50–59	+	+	28.9	222G (+)	Lung, trachea, upper respiratory tract
2	F	40–49	+	+	23.3	222D (−)	Lung, trachea, upper respiratory tract
3	F	11–20	+	+	30.1	222D (−)	Trachea, upper respiratory tract, nasopharyngeal airway
4	M	20–29	+	+	35.4	222D (−)	Trachea
5	F	1–10	+	+	16.3	222D (−)	Nasopharyngeal airway
6	M	11–19	+	+	25.8	222D (−)	Nasopharyngeal airway
7	F	30–39	+	+	26.9	222G (+)	Lung, nasopharyngeal airway
8	M	20–29	+	+	26.3	222G (+)	Lung, trachea
9	F	60–69	+	+	36.5	222D (−)	Nasopharyngeal airway
10	M	50–59	+	+	35.4	222D (−)	Nasopharyngeal airway
11	F	50–59	+	+	27.6	222D (−)	Nasopharyngeal airway
12	M	40–49	+	+	23.9	222D (−)	Lung, nasopharyngeal airway
13	M	30–39	+	+	21.6	222G (+)	Lung, nasopharyngeal airway
14	M	40–49	+	+	24.1	222G (+)	Lung
15	M	50–59	+	+	32.7	222G (+)	Nose, bronchus

M, male; F, Female.

### 3.3 Detection of influenza‐specific cells in the lung tissue

**Figure 1:**
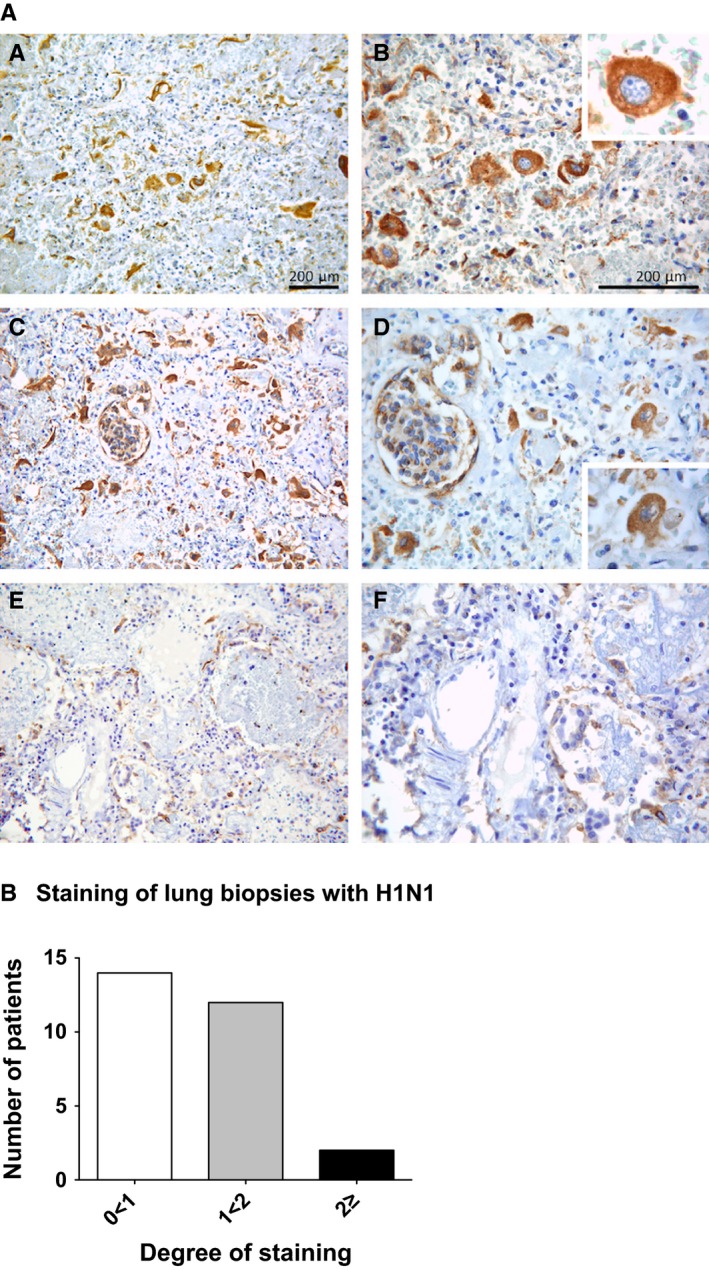
Detection of influenza A H1N1‐specific cells locally at site of inflammation in the lung tissue of fatal Norwegian cases. (A) Immunohistochemical staining was performed on paraffin‐embedded formalin‐fixed lung tissue sections to identify influenza‐specific cells (brown) at site of inflammation. These were observed sporadically throughout the tissue (A, B), and were also detected in close proximity to and inside alveolar structures (C, D). Some cases had less (E, F) influenza‐specific cells at the site of inflammation than others (A, B and C, D). (B) The degree of H1N1 staining in the lung tissue was evaluated. Depending on the level of staining, either number 0, 1 or 2 was assigned for each category during assessment, where 0 was considered negative, 1 was regarded positive and 2 represented strongly positive. The *x*‐axis illustrates the degree of staining, while the *y*‐axis indicates the number of patients

Immunohistochemical staining was performed on paraffin‐embedded formalin‐fixed lung tissue sections to identify influenza‐specific cells. This visualisation provided us with a more detailed understanding of the viral expression pattern at the site of inflammation, which was prominent, both in close proximity to and inside alveolar structures (Fig. 1A). Moreover, the degree of A(H1N1)pdm09 staining in the lung tissue was evaluated (Fig. 1B). Two tissue sections were evaluated from each subject, where the mean score value from both sections was calculated and illustrated. Most of the patients showed a staining score of 0<1 (14 subjects) and 1<2 (12 subjects), while only two subjects had a staining score of 2. Although no direct association between disease severity and staining intensity could be observed, the high number of staining score 0 could be a result of tissue destruction as a consequence of disease severity.

## 4 Discussion

Since the outbreak of the novel A(H1N1)pdm09, post‐pandemic studies have described the clinical aspects of this virus.^23^, ^24^, ^25^ Several host factors and underlying conditions have been associated with disease severity.^9^, ^14^, ^15^, ^16^, ^20^, ^21^, ^22^ These include pneumonia, obesity, pregnancy, in addition to effects of vaccines and neuraminidase inhibitors that were used during the pandemic.^23^, ^24^, ^25^ Kilander et al.^29^ reported a novel mutation in the major surface glycoprotein HA) of A(H1N1)pdm09, namely HA D222G, which was associated with a severe clinical outcome in Norwegian patients.^30^ The mutation was found with considerable frequency in fatal and severe cases (11 of 61 cases), but was not observed in any of the 205 mild cases included in their study. Other subsequent studies have found this mutation at considerable frequency in fatal and severe cases.^26^, ^28^, ^29^, ^30^ In this study, sequence analysis of the A(H1N1)pdm09 virus showed that many of the genetic markers associated with virulence were not present. Moreover, the most severe clinical pandemic cases also showed severe pathological damage in the lungs, where in susceptible individuals inflammation of the alveolar walls resulted in DAD. Therefore, we further investigated whether there was an association between the genetic viral HA D222G mutation and the expression of the A(H1N1)pdm09 virus locally at the site of inflammation in the lung tissue. In this study, we performed a thorough characterisation of nineteen of the fatal Norwegian pandemic cases. The increased morbidity encountered with the virus is related to infection of cells in the upper and lower airways and ultimately resulting in DAD.^37^ Here, we identified possible genetic viral mutations, while additionally visualising the expression of the A(H1N1)pdm09 virus locally in the lung tissue of these subjects.

Our results demonstrated that of the 19 fatal cases that were hospitalised during the pandemic in Norway, the course of infection was ≤14 days in 79% of these cases. Neuraminidase inhibitors were administered to 47%. Compared with no treatment, neuraminidase inhibition therapy has previously been associated with a reduction in mortality risk regardless of timing.^25^ Interestingly, evaluation of the lung tissue from our fatal cases revealed that 11 individuals had DAD (78%) and 4 of these patients were seemingly healthy and without apparent pre‐existing illness.^38^ With a short overall course of illness that lasted a range of 2–40 days, all 4 previously healthy individuals had a surprisingly short course of infection of ≤11 days (Table 1).

As observed previously, obesity was also a prominent contributor to disease severity during the pandemic in these 19 fatal cases,^39^ 12 had a BMI that ranged from 29 to 55, and an overall course of illness that lasted a range of 7–40 days. Interestingly, 6 of the obese subjects showed DAD in their lung tissue, 3 of which belonged to the healthy group of individuals with no apparent pre‐existing illness (Table 1). This suggests that a high BMI may influence the susceptibility of developing DAD in healthy individuals.

All subjects in this study were positive by real‐time PCR for A(H1N1)pdm09 virus. The viral mutation HA D222G was observed in 6 of 15 cases where the genotype in this position could be ascertained, while the other nine patients possessed the wild‐type 222D.^30^, ^40^, ^41^ Interestingly, all four subjects who developed DAD and had no pre‐existing illness expressed the HA D222G viral mutation in their lung tissue (Table 2). This suggests that, similar to obesity, the expression of the HA D222G viral mutation at the site of inflammation predisposes to viral pneumonia and development of DAD with an associated increased mortality rate. Whereas the likelihood of occurrence of this and other mutations may increase the duration of infection, the duration of illness before death was found to be comparably short for these cases of HA D222G in previously healthy individuals. Due to the nature of the study, only fatal cases were included and further studies comparing the whole viral genome sequence between mild and fatal cases may shed light on the role of the virus in mortality.

The identification and visualisation of A(H1N1)pdm09‐specific cells on paraffin‐embedded formalin‐fixed lung tissue sections from the subjects, using immunohistochemistry, provided us with a more detailed understanding of the viral expression pattern at the site of inflammation.^42^ These influenza‐specific cells were prominent, both in close proximity to and inside alveolar structures in the tissue (Fig. 1A). Moreover, evaluating the degree of H1N1 staining in the lung tissue and the distribution of the staining intensity revealed that 74% of the patients had a relatively low staining score of 0<1 (14 subjects), while only two subjects had a staining score of 2 (Fig. 1B). No direct association between disease severity and staining intensity was observed in these 19 cases. We could not, however, ignore the possibility that both differences in disease severity and post‐mortem procedures have influenced the quality of the material examined, which may explain the rather high number of staining score 0. Virus‐induced inflammation probably causes lung pathology, but tissue destruction can also be a result of immune‐mediated killing of virus‐infected. Therefore, the role of immune cells, particularly cytotoxic T cells, in lung pathology should be evaluated in future studies.

In conclusion, our results show a possible association in fatal Norwegian cases from the A(H1N1)pdm09 pandemic between mortality and the factors obesity, underlying illness, and the viral mutation HA D222G. Furthermore, the visualisation of influenza‐specific cells in lung tissue sections from these same individuals provided us with a deeper understanding of the viral expression pattern at the site of inflammation. Combined, our results suggest that in many cases both underlying conditions and host factors in the infected individuals were fundamental for disease outcome, rather than the virus itself. This study expands our understanding of determinants for the clinical outcome of pandemic influenza, which could in turn guide future treatment.

## Appendix 1

### Norwegian lung pathology group

Erik Heyerdahl Strøm, Department of Pathology, Oslo University Hospital Rikshospitalet; Torleiv O. Rognum, Institute of Forensic Medicine, Oslo University Hospital Rikshospitalet; Jan Mæhlen, Department of Pathology, Oslo University Hospital Ullevål; Glenny Cecilie Alfsen, Department of Pathology, Akershus University Hospital, Akershus County; Trond Viset, Department of Pathology and Medical Genetics, St. Olavs Hospital, Trondheim; Ingjerd Lien Kvelstad, Department of Pathology, Sykehuset Innlandet Hospital Trust, Lillehammer; Inge Morild, Gade Laboratory of Pathology, Department of Clinical Medicine, University of Bergen.
